# Antipyrine and Thallin

**Published:** 1887-01

**Authors:** John A. Robison

**Affiliations:** Professor of Materia Medica and Therapeutics, Woman’s Medical College; Lecturer on Materia Medica, Rush Medical College; 428 Washington Boulevard


### CONSIDERATION OF ANTIPYRINE AND THALLIN AS ANTIPYRETICS.

*By* JOHN A. ROBISON, A.M., M.D., *Professor of Materia Medica and Therapeutics, Woman's Medical College; Lecturer on Materia Medica, Rush Medical College:*

[Read before the Chicago Medical Society on December 6th, 1886.]

The therapeutical actions of antipyrine and thallin, as detailed in the briefest possible manner in this paper, have been selected from the observations of a large number of clinicians. The entire literature on the subject has been carefully examined and the true therapeutical actions of these drugs epitomized.

Antipyrine is an artificial alkaloid discovered by Ludwig Knorr, of Erlängen, and obtained by the reaction of acetic ether on aniline, or by oxidation of chinoline. Antipyrine is in the form of a white crystalline powder, of a faint aromatic odor and insignificant bitterness of taste, and is freely soluble in water and alcohol. The solutions are neutral, in reaction, and can be used hypodermically. The dose of the drug is, by the mouth, fifteen to thirty grains, hypodermically, half these quantities. From sixty to ninety grains must be given to procure the therapeutical effects, and it is better to give the drug at intervals of an hour until the desired effects are produced. As far as possible, we will review the actions of this drug on the various systems of the body, in regular order.

*Digestive System.*—Its taste is slightly bitter and can easily be disguised, therefore it seldom produces nausea or vomiting on first administration. Large doses have sometimes an astringent effect on the intestinal tract, producing constipation, and checking diarrhœa and intestinal hemorrhage. It is readily absorbed, and is freely eliminated by the skin, kidneys and bowels. During the process of elimination by the skin it produces profuse perspiration, and in some patients a peculiar eruption. About ten per centum of patients experience this eruption.

*Circulatory System.*—Antipyrine in large doses dilates the veins and capillaries, thus increasing heat radiation. It has, contrary to general belief, a *tonic action* upon the heart, and not only strengthens the auricles but increases slightly the blood-pressure. In no respect does antipyrine injuriously effect the blood or muscular tissues. The frequency of the pulse generally varies with the changes of temperature, *i. e.*, falls when the temperature descends, and rises with the ascent of the latter.

*Respiratory System.*—Nil.

*Cerebral and Nervous System.*—A full dose of antipyrine induces a sense of drowsiness and exhiliration. Fatigue is displaced by a feeling of well-being. Dr. J. B. White, of New York, says in the *Medical Record*, September 11th, that "the drug when given in masterful doses will relieve headache whenever present, whether resulting from disordered digestion, disturbance of the menstrual functions, loss of sleep, undue mental effort, or even uræmia; and also it possesses reliable prophylactic virtues against attacks of cranial neuralgia." In the delirium of pneumonia and fevers, the administration of antipyrine is often followed by periods of lucidity.

*Action on Tissues.*—The action of antipyrine in reducing temperature is perhaps the one most interesting to us. We have already mentioned the fact that it increases the circulation of the blood through the veins and capillaries and promotes perspiration, and thus probably reduces the temperature by increased radiation of heat; and yet this will not account for the marked reduction of temperature which follows the administration of large doses of the drug at frequent intervals. Umbach's experimental studies in Nencke's laboratory at Berne demonstrated that antipyrine lessened the amount of elimination of nitrogenous waste both by the general and respiratory systems. Hence he concluded that this drug, like quinine, produces apyrexia chiefly by limiting the oxidation of tissues. Therefore it is indicated when rapid oxidation of the tissues gives rise to an excessively high temperature. In such cases the nutritive processes are arrested until the excessive waste of tissue is controlled. In these cases apyrexia should be induced as soon as possible. The apyrexia produced by antipyrine varies in its rapidity and intensity according to the age, sex and constitution of the patient, the time and mode of administration, and the nature and stage of the disease. The younger the patient and the more debilitated, the more promptly will the antipyretic effect be obtained. If the action of the drug is obtained during a spontaneous fall of the temperature, a condition resembling collapse may be produced, with the temperature below normal. The effect is heightened by the use of cold baths and the administration of other antipyretics. Fifteen grains given at intervals of one hour will generally cause a fall of temperature from one to two degrees. In typhoid fever, during the first stages of the disease, it frequently requires the administration of seventy-five grains to produce an apyrexia lasting only three or four hours, while in the later stages of the disease forty-five to sixty grains will cause the temperature to fall to 99° or 100° F. for a period of ten to twelve hours. Much larger doses of the drug produce no more profound effects.

The question arises, does antipyrine cut short the duration of any of the febrile diseases in which it is given?

In 130 cases of typhoid fever in which I have exhibited the drug, the average length of time of treatment was twenty-one and one-half days. The least number of days of treatment was five; the largest, fifty. Of course these figures do not represent the number of days the patients were sick. My opinion is that the drug neither lengthens nor lessens the duration of the diseases in which it is given. But in cases of excessively high temperature, where it is *per se* a danger, I believe antipyrine conserves the vital forces and gives a better chance for recovery, although the drug may not hasten covalescence. In these cases I believe it to be the most reliable, safest, and the least objectionable of any antipyretic at our command. In the case of C. H., whose fever-chart is here presented, during the twenty days antipyrine was given there were 226 hours in which the temperature was on the down grade. Nearly half the time there was lessened tissue-waste, as evidenced by the lowering of the temperature. The average duration of the apyrexia was four hours and six minutes. There were no chills or other disturbances.

*Pneumonia and other febrile diseases.*—In pneumonia, acute articular rheumatism, malarial fever and phthisis this drug controls the temperature without producing the dangerous symptoms that often follow the administration of large doses of quinine, kairin, salicylic acid, or thallin. In the acute articular rheumatism of children, with endocarditis, I have had good results by administering it in combination with alkaline treatment. The only report of a case of failure to produce apyrexia I have noticed, is a case of sun-stroke reported in the *Medical Record*, September 25, 1886.

THALLIN.

Thallin is another artificial alkaloid, of a fawn color, bitter taste, and sticky, disagreeable odor. It may be given in doses of one-half to three grains, at intervals of one or two hours until apyrexia is produced.

*Apyrexia.*—It lowers the temperature even more readily than antipyrine, and often succeeds when antipyrine fails.

*Circulatory System.*—In small doses thallin increases the rate of the heart's action, but in larger doses decreases it, enfeebles the circulation, and, if the dose be lethal, the heart's action is arrested in diastole. Under thallin, venous blood and the walls of the veins become darker in hue than normal. Therefore, this drug should not be used in infectious diseases on account of thallinization of the blood.

*Chills.*—The apyrexia produced by thallin is almost always accompanied by severe chills. In the case of J. J., whose temperature chart is also presented, in which thallin was used in one to three grain doses for six days, six chills occurred, although antipyrine was used 40 days without a chill occurring. The chill was always accompanied by a sudden rise in temperature; it once rose as high as 106.2° F.

*Pulse, Respiration.*—The pulse becomes feeble, irregular and quick, and the respirations shallow in a number of instances when thallin is given in therapeutic doses.

In comparing the antipyretic actions of these new fever-reducing drugs, I think all observers agree that antipyrine is efficient and safe, whereas thallin must be used with a great deal of discrimination.

428 Washington Boulevard.

